# Spectral-selective light detection and ranging at 1550 nm using tandem colloidal quantum dot photodiodes

**DOI:** 10.1038/s41377-026-02244-2

**Published:** 2026-08-20

**Authors:** Junrui Yang, Jing Liu, Chengjie Deng, Shuaicheng Lu, Hao Ding, Xing Zhou, Long Hu, Xinzheng Lan, Jianbing Zhang, Xing Yang, Yihua Hu, Jiang Tang, Liang Gao

**Affiliations:** 1 Wuhan National Laboratory for Optoelectronics (WNLO) and School of Optical and Electronic Information, Huazhong University of Science and Technology, Wuhan, China; 2 State Key Laboratory of Pulsed Power Laser Technology, Hefei, China; 3 Optics Valley Laboratory, Wuhan, China; 4 Wenzhou Advanced Manufacturing Technology Research Institute of Huazhong University of Science and Technology, Wenzhou, China; 5 JFS Laboratory, Wuhan, China; 6 Shenzhen Huazhong University of Science and Technology Research Institute, Shenzhen, China; 7 School of Materials Science and Engineering, Huazhong University of Science and Technology, Wuhan, China; 8 School of Materials Science and Engineering, The University of New South Wales, Sydney, NSW Australia

**Keywords:** Optical sensors, Imaging and sensing

## Abstract

Light detection and ranging (LiDAR) systems enable three-dimensional (3D) imaging for emerging applications in autonomous driving and mixed reality. The operational wavelength of LiDAR detectors is transitioning from 905 nm to 1550 nm, leveraging the higher safety power threshold and superior coherence of 1550 nm lasers. Colloidal quantum dots (CQDs) photodiodes have shown good performance and low cost for two-dimensional (2D) imaging covering 1550 nm, however, been faced two main challenges, including poor spectral selectivity and slow response speed as a LiDAR detector. Here, we introduce a tandem PbSe CQDs photodiode that functions as an integrated photonic-electronic link, overcoming both challenges by synergistically merging optical pre-processing and electronic post-processing within a single structure. The designed tandem structure leverages a synergistic combination of RC time constant suppression and charge collection narrowing, yielding a record-fast response time of 1.01 ns among the reported CQD devices and a 13-fold suppressed external quantum efficiency (EQE) in the visible range compared to single-junction devices. We further showcase a spectral-selective LiDAR operating at 1550 nm using the tandem CQDs photodiodes, achieving a centimeter-level spatial resolution while maintaining 97.9% of the signal-to-noise ratio under strong background interference.

## Abstract (web summary)

A tandem colloidal quantum dot photodiode is engineered as an integrated photonic–electronic link, enabling optical pre-processing and electronic post-processing for spectrally selective, 1.01 ns 1550-nm LiDAR.

## Graphical abstract

**Figure:**
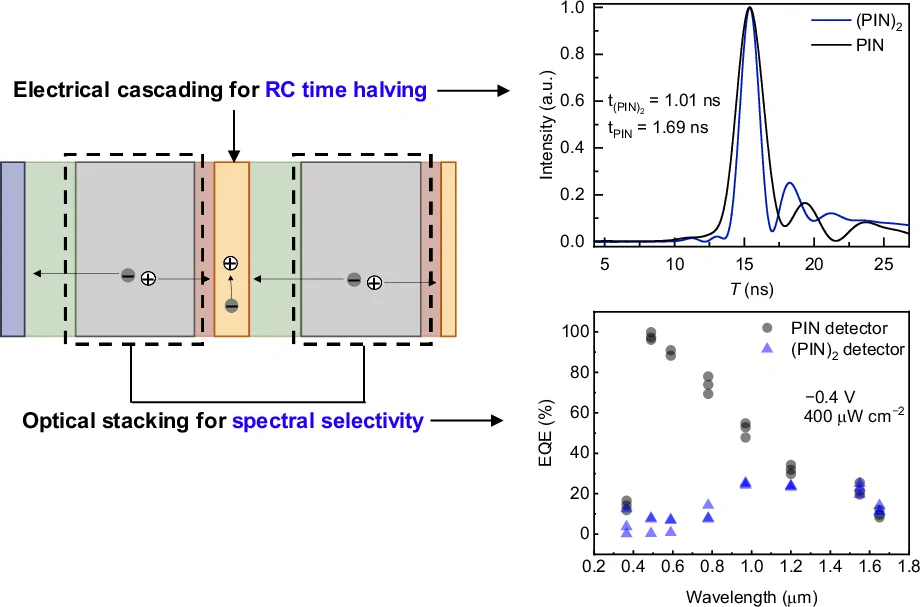

## Introduction

Light detection and ranging (LiDAR) systems have been widely employed in applications such as autonomous driving^1–3^, mixed reality^4^, medical imaging^5^, and industrial production monitoring^6–11^. They have two important parts of a short-pulse laser and a detector, in which the detector measures the time of the short-pulse laser to scatter back from a target object. The LiDAR system acquires the distance of a target object by multiplying the light speed with the measured time and scans a target scene to construct a three-dimensional (3D) image. Nowadays, the LiDAR detectors commercially rely on silicon photodiodes working at 905 nm or InGaAs photodiodes working at 1550 nm. The working window of LiDAR detectors tends to transfer from 905 nm to 1550 nm, benefiting from higher laser safety power for eyes at 1550 nm in the national standard^12^ and superior coherence of 1550 nm lasers demanded in the emerging frequency-modulated continuous-wave (FMCW) LiDAR^13,14^. However, the InGaAs photodiodes operating at 1550 nm suffer from significantly increased cost and complicated processes for further LiDAR detector array^15–18^, which are unaffordable in consumer electronics and autonomous driving.

Solution-processed colloidal quantum dots (CQDs) have drawn significant interest for infrared detection due to their high absorption coefficient^19^, multi-exciton generation^20^, and finely tunable bandgap^21^. The CQDs photodiodes have customized response cutoffs over 1550 nm and high external quantum efficiency (EQE) at 1550 nm based on quantum confinement effects^22,23^. The CQDs photodiodes enable monolithic integration with silicon-based readout circuits to form an infrared imaging array, of which the cost is over two orders of magnitude lower than InGaAs imaging array^24,25^. The CQDs imaging arrays have shown high resolution and broad response range for capturing two-dimensional (2D) images. However, on the road from 2D imaging to 3D LiDAR, the CQDs photodiodes have been stuck by two big challenges including inadequate spectral selectivity against visible-light interference and slow response speed.

Although CQDs inherently possess an exciton absorption peak at 1550 nm due to the quantum confinement effect^26,27^, their absorption coefficient in the visible range is approximately 4-fold higher than at the target 1550 nm as shown in Fig. S1. This pronounced visible absorption leads to a substantially stronger photoresponse to background interference than the desired 1550 nm LiDAR signal, severely degrading the signal-to-noise ratio of the system. Even with optical filters employed to suppress sunlight interference, the scattered-back light intensity of the 1550 nm short-pulse laser remains 24.5 times weaker than the filter-leaked sunlight under AM1.5 G, as detailed in Note S1 and Fig. S2. The higher visible light absorption coefficient of CQDs than at 1550 nm still has certain impacts on the signal-to-noise ratio of the LiDAR system, as shown in Fig. S3.

**Fig. 1:**
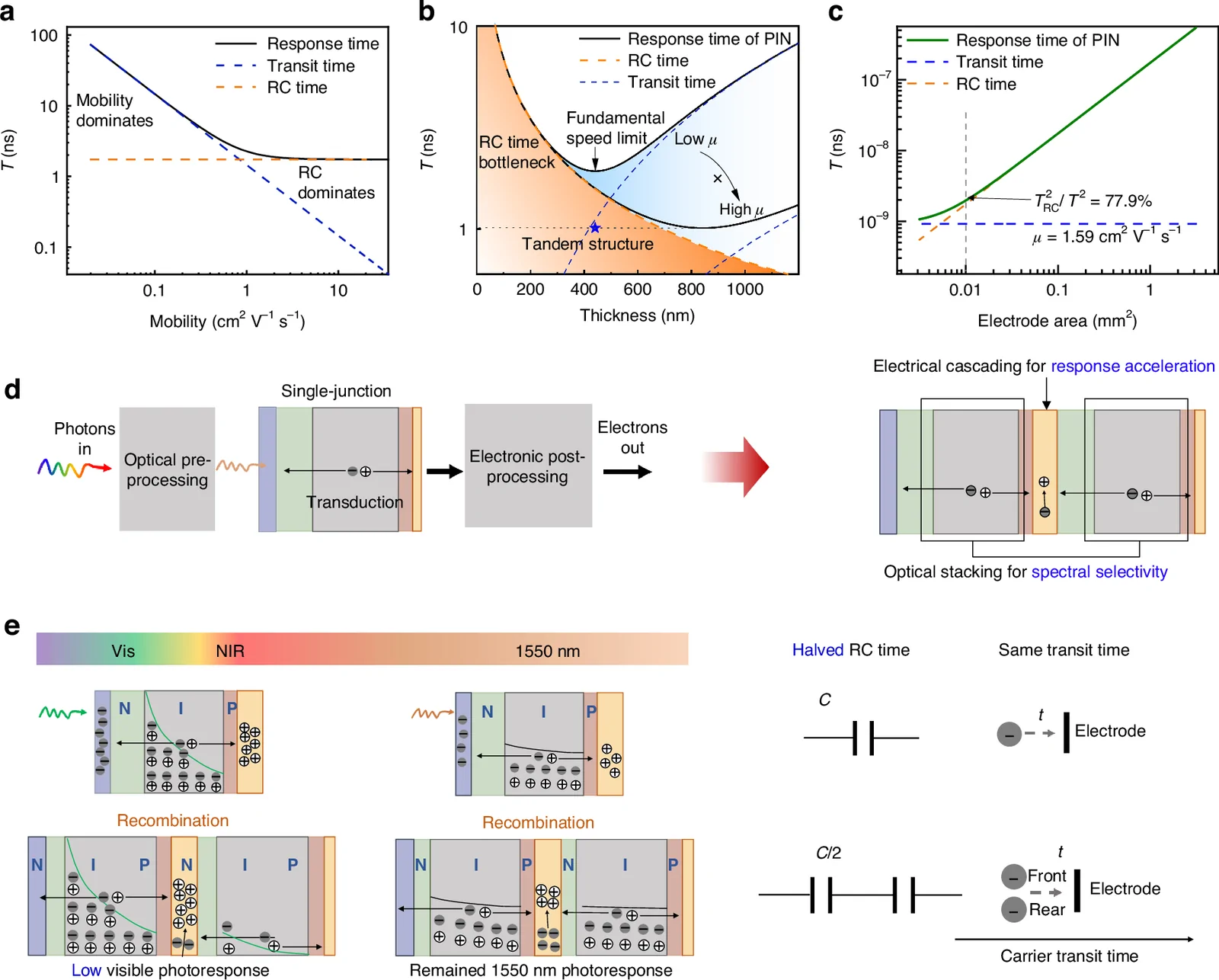
Working principles and theoretical performance of tandem photodiodes for LiDAR. **a** Theoretical response time and its components as a function of charge carrier mobility. **b** Calculated response time versus depletion region thickness. **c** Predicted response time and constituent times for a single-junction device across varying active areas. **d** Conceptual diagram of the tandem photodiode as an integrated photonic-electronic link, enabling optical pre-processing and electronic post-processing. **e** Operating mechanism of the tandem structure for spectral selectivity and enhanced response speed

Besides, the response time of CQDs photodiodes needs to be shortened for higher ranging accuracy of LiDAR system^28,29^. The response time of CQDs photodiodes is composed of the transit time governed by carrier mobility and the RC time determined by junction capacitance^30^ (Note S2). The speed bottleneck crosses over from transit time to RC time as mobility surpasses ~1 cm^2 ^V^−1^ s^−1^ (Fig. 1a). Conventional strategies to enhance response speed have focused on reducing device area to minimize RC time, coupled with developing high-mobility CQDs films^22,31–35^. As the two primary strategies for minimizing junction capacitance, depletion layer thickness optimization and device area scaling encounter fundamental bottlenecks. Optimizing the depletion layer thickness involves a fundamental trade-off between capacitance and transit time, resulting in a fundamental speed limit (Fig. 1b). Aggressive scaling of the device area is ultimately constrained by the optical diffraction limit (the system’s Airy disk) and leads to a diminished light-collection area, adversely affecting the LiDAR system’s signal-to-noise ratio. Furthermore, as revealed in Fig. 1c, even at a small area of 0.01 mm^2^, the RC time still accounts for 77.9% of the total response time in a single-junction device, indicating that the RC bottleneck is not easily circumvented by scaling alone.

We introduce a tandem CQDs photodiode architecture (called (PIN)_2_ device) as a structural innovation that fundamentally addresses both limitations of CQDs-based LiDAR detectors. This tandem architecture functions as an integrated photonic-electronic link, concurrently performing optical pre-processing and electronic post-processing to enhance both spectral selectivity and response speed (Figs. 1d and S4). The operational mechanism for spectral selectivity and response time is illustrated in Fig. 1e. In conventional single-junction CQDs photodiodes (PIN devices), the higher absorption coefficient in the visible range compared to 1550 nm results in significantly stronger response to background interference than to the target LiDAR signal. In contrast, the tandem architecture comprising two identical photodiodes exploits this absorption disparity for spectral filtering. For visible wavelengths, the higher absorption coefficient leads to shorter penetration depth, creating unbalanced light flux between the front and rear photodiodes. Since both sub-junctions are connected in series and experience identical electric fields, they must generate equal current. The photocurrent mismatch caused by unequal carrier generation leads to cancellation of excess carriers from the front photodiode, thereby effectively suppressing the visible-light photoresponse through the charge collection narrowing mechanism^36,37^. Conversely, at the target 1550 nm wavelength, the longer penetration depth ensures balanced carrier generation across both junctions, preserving the photoresponse comparable to single-junction devices. This built-in spectral filtering capability provides a crucial supplementary defense against residual interference that bypasses external optical filters, establishing a comprehensive approach to background rejection distinct from merely shifting the response peak into atmospheric windows^38–40^.

The tandem architecture introduces a structural degree of freedom that fundamentally transcends the conventional speed limitations of single-junction photodiodes. As revealed by equivalent circuit analysis (Note S3 and Fig. S5), the tandem configuration achieves minimal capacitance when the two constituent photodiodes possess matched capacitance. This creates a distinct advantage over conventional scaling approaches. In a single-junction device, doubling the CQDs layer thickness to reduce capacitance would simultaneously double the carrier transit path and halve the electric field intensity, resulting in a fourfold increase in transit time. In contrast, the tandem architecture strategically decouples these competing factors: it achieves approximately halved junction capacitance through series connection while maintaining the same transit length as a single junction. This enables a fundamental reduction in RC time constant without compromising transit time, breaking the inherent trade-off that limits conventional device optimization. Consequently, the tandem photodiodes achieve a systematic enhancement in response speed, primarily governed by the approximately twofold reduction in RC time constant.

In this work, we demonstrate tandem PbSe CQDs photodiodes specifically designed for 1550 nm LiDAR applications. This architectural innovation aligns with the industry trend toward longer operational wavelengths while offering a viable pathway to low-cost 3D detector arrays. Through careful optimization of the device structure and layer thicknesses, our tandem photodiodes simultaneously address the dual challenges of response speed and spectral selectivity. The resulting tandem CQDs photodiodes achieve a record-fast response time (1.01 ns) among the reported CQDs devices, a large linear dynamic range (>99.6 dB), and a high detectivity (2.29 × 10^11^ Jones) at 1550 nm. Remarkably, the tandem architecture provides a 13-fold suppressed EQE in the visible range (reduced to 7%) while maintaining uncompromised EQE at the target 1550 nm (25.06%). We further validate the system-level performance by implementing a spectral-selective LiDAR system that achieves centimeter-level spatial resolution while maintaining 97.9% signal-to-noise ratio under strong background interference. These results establish tandem CQD photodiodes as a promising technology for next-generation, cost-effective 1550 nm LiDAR systems.

## Results

To minimize the carrier transit time in our photodiodes, we selected PbSe CQDs with an excitonic absorption peak at 1550 nm as the light-absorbing material, leveraging their superior carrier mobility compared to PbS CQDs^41^. The PbSe CQDs films were fabricated using the widely established mixed halide ligand-exchange method^42–44^. Comprehensive characterization of charge transport properties through multiple techniques—including field-effect transistor (FET) measurements, terahertz (THz) spectroscopy, and space-charge-limited-current (SCLC) analysis (Fig. S6)^45^—reveals a carrier mobility range of 0.21 to 3.65 cm^2 ^V^−1^ s^−1^ for halide-capped PbSe CQDs films. This represents an improvement of approximately two to three orders of magnitude over conventional PbS CQDs films^41,46^. Electrical characterization in N_2_ atmosphere confirmed n-type doping behavior with carrier concentrations of 10^16^–10^17 ^cm^−3^ (Fig. S7). Exposure to ambient air resulted in a significant reduction in carrier concentration to approximately 10^15 ^cm^−3^, accompanied by a two-order-of-magnitude decrease in mobility (Fig. S8), attributable to the detrimental effects of H_2_O/O_2_ interaction with both the CQDs surface and the SiO_2_ gate^47–50^. All fabrication processes were conducted in a N_2_ atmosphere to prevent surface oxidation and preserve the optimal electronic properties of the PbSe CQDs.

The conventional structure of CQDs photodiode comprises a ZnO electron transport layer (200 nm), a halide-capped PbSe CQDs absorption layer (400 nm), and an ethanedithiol (EDT)-treated PbS CQDs hole transport layer (20 nm)^51,52^. Significant rectification behavior and efficient photogenerated carrier transfer are observed at the junction between p-type EDT-PbS CQDs (bandgap ~1.4 eV) and n-type halide-PbSe CQDs (bandgap ~0.8 eV), as evidenced in Figs. S9 and S10. But this rectifying behavior is absent at the interface between n-type ZnO and n-type PbSe CQDs, leading to suboptimal device performance. To address the poor photoresponse observed in the simple ZnO/halide-PbSe CQDs/EDT-PbS CQDs structure, we introduced an intermediate grading layer of halide-capped PbS CQDs (bandgap ~0.95 eV). This strategic insertion significantly improves the interfacial compatibility between the EDT-PbS and halide-PbSe layers, facilitating more efficient charge carrier extraction and enhancing the overall photoresponse (Fig. S11). The optimized PbSe CQD photodiode, featuring this tailored band alignment (Fig. S12), demonstrates excellent electrical characteristics including low dark current, high rectification ratio, and substantially improved photoresponse, as detailed in Fig. S13.

**Fig. 2:**
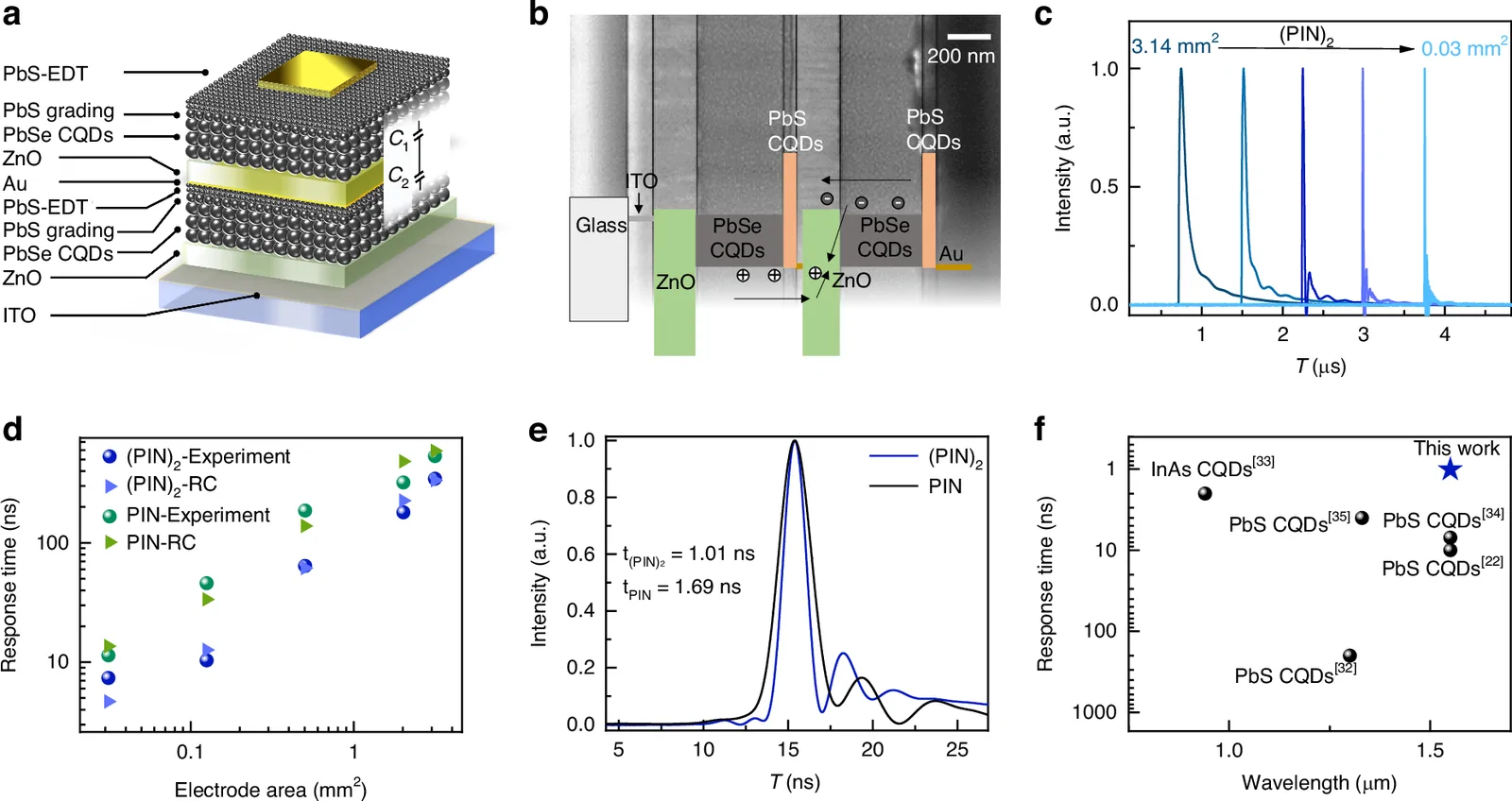
Device structure and pulse laser responses of tandem CQDs photodiodes. **a** Schematic diagram and **b**, Cross-sectional scanning electron microscope (SEM) image and energy band alignment of tandem photodiodes. **c** Pulse laser responses of tandem photodiodes with active areas varying from 3.14 to 0.03 mm^2^ at zero bias. **d** Measured response time and calculated RC time of single photodiode and tandem photodiodes with active areas varying from 3.14 to 0.03 mm^2^. **e** Measured response time of single photodiode and tandem photodiodes with an active area of 7.85 × 10^−3^ mm^2^. **f** Statistics of response times of the reported fast CQDs photodiodes and tandem photodiodes

Two identical PbSe CQDs photodiodes are connected in series through a 1 nm Au intermediate layer (Fig. 2a). The substrate is indium tin oxide (ITO) glass with over 70% transmittance at 1550 nm. The top Au electrodes are patterned by a shadow mask to define various active areas (Fig. S14). Along the direction of incident light, we designate the two stacked photodiodes as the front and rear junctions, both fabricated using identical processes as described in the Methods section. The cross-sectional SEM image in Fig. 2b confirms the precise thickness matching between corresponding layers in the front and rear junctions. This structural symmetry ensures equal capacitance between the two sub-cells, a critical condition for minimizing the overall RC time of the tandem photodiodes through the series capacitance reduction mechanism.

Given the competing dependence of both transit time and RC time on the PbSe CQDs film thickness, we systematically investigated this critical parameter to identify the optimal device configuration. The thickness of the PbSe CQDs films was precisely controlled by varying the concentration of CQDs inks, with exact values quantified using a step profiler (Table S1). Fig. S15 presents a comprehensive analysis of both calculated and measured response times as functions of electrode area and CQDs film thickness. The calculated response times, derived using carrier mobility values obtained from the SCLC method, show excellent agreement with experimentally determined values across all device geometries and thickness variations. This close correspondence validates the relevance of SCLC-derived mobility parameters for predicting the performance of actual photodiode devices and confirms the accuracy of our device modeling approach.

We conducted a systematic comparison between single-junction and tandem photodiodes fabricated with the optimized PbSe CQDs film thickness of 400 nm. The transient responses to pulsed laser illumination, presented in Figs. S16 and 2c, demonstrate a consistent improvement in response speed with decreasing active area from 3.14 to 0.03 mm^2^. The measured response times are acquired as the times that the pulse responses fall from 90% to 10% and are nearly linear to the device active area, as shown in Fig. 2d. The response time of the tandem photodiodes is about half of that of the single photodiode with the same active area. This performance enhancement directly correlates with the reduction in RC time constant, as confirmed by capacitance-frequency (*C*-*f*) curves in Fig. S17. The close correspondence between the trends in response time and RC time constant provides compelling evidence that the device speed in these high-mobility PbSe CQDs photodiodes is predominantly limited by the RC time constant across the investigated active areas.

Further characterization of devices with a minimal active area of 7.85 × 10^−3^ mm^2^ reveals the ultimate performance limits of our architecture. As shown in Figs. 2e and S18, the single PbSe CQDs photodiode achieves a response time of 1.69 ns, while the tandem configuration establishes a new record of 1.01 ns—the fastest response time reported to date for CQDs photodiodes, as benchmarked against state-of-the-art devices in Table S2 and Fig. 2f^22,32–35^. Our tandem device also occupies the most advanced position in the response time-active area landscape (Fig. S19). The response time of the tandem device varied with pulse laser intensity, is shown in Fig. S20. When the laser intensity ranges from 0.3 mW cm^−2^ to 4.6 mW cm^−2^, the response times of the tandem device remain almost the same due to the efficient extraction of photo-generated carriers. This intensity-independent behavior demonstrates highly efficient extraction of photogenerated carriers and robust device operation under diverse working conditions, confirming the practical suitability of our tandem photodiodes for real-world LiDAR applications.

The operational principle of the tandem photodiodes relies on the strategic management of photocurrent matching between the front and rear junctions, which is governed by their wavelength-dependent absorption characteristics. In order to suppress the photocurrent mismatch at the target 1550 nm, we used the basic optical parameters of PbSe CQDs (Fig. S22) for light field construction in the tandem photodiodes. The finite-difference time-domain (FDTD) model of the tandem photodiodes in Fig. S23 reveals that the front and rear junctions have almost the same absorption at 1550 nm when the thickness of CQDs layer is around 400 nm due to the reflection of the top Au electrode. In contrast, within the visible spectrum, the front junction exhibits significantly stronger absorption than the rear junction, attributable to the substantially higher extinction coefficient of PbSe CQDs at shorter wavelengths. This engineered absorption disparity creates a wavelength-selective response: in the visible range, the pronounced photocurrent mismatch between junctions effectively suppresses unwanted background response through the charge collection narrowing mechanism, while at 1550 nm, the balanced absorption ensures optimal photocurrent matching and maximizes the target signal response.

**Fig. 3:**
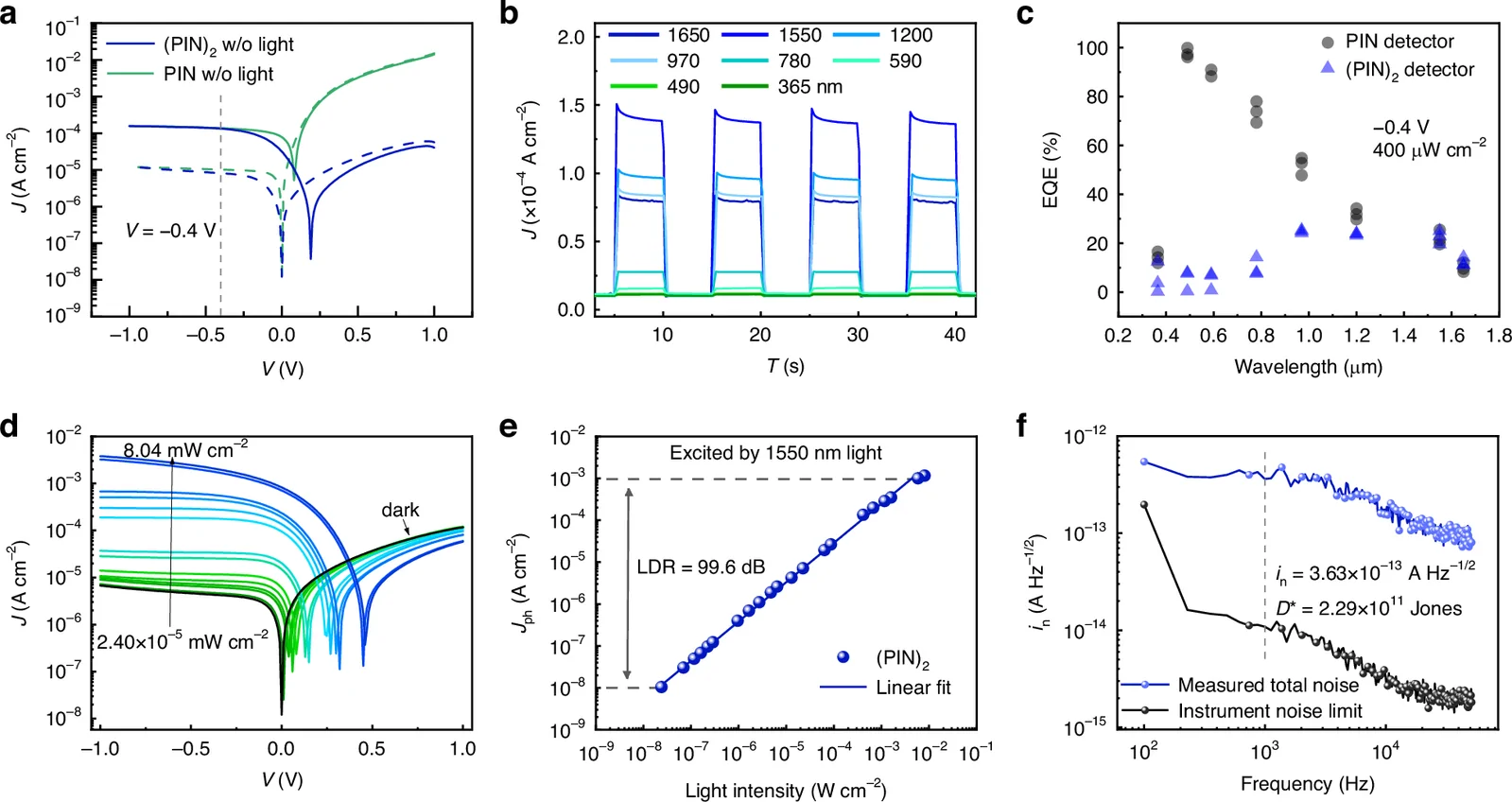
Device performance and anti-interference capability of tandem CQDs photodiodes. **a** Current density-voltage (*J*-*V*) curves of single photodiode and tandem photodiodes under dark condition or 1550 nm light with a power density of 400 μW cm^−2^. **b** Modulated light responses of tandem photodiodes at −0.4 V under illumination with different wavelengths (365–1650 nm) and the same power density of 400 μW cm^−2^. **c** Broadband EQE spectra of single photodiode and tandem photodiodes at −0.4 V acquired from *J*-*V* curves (Fig. S21). **d** *J*-*V* curves of tandem photodiodes illuminated by 1550 nm light with varied power densities from 2.40 × 10^−5^ to 8.04 mW cm^−2^. **e** Linear dynamic range of tandem photodiodes. **f** Noise current spectrum of tandem photodiode

To experimentally validate the FDTD simulation predictions, we systematically characterized the photo-responses to 1550 nm light of tandem photodiodes with the thicknesses of PbSe CQDs layers ranging from 75 to 560 nm (Fig. S24). The responsivity exhibits a pronounced thickness dependence, increasing from 0.05 A W^−1^ at 75 nm to a maximum of 0.23 A W^−1^ at the optimized 400 nm thickness, then sharply declining to 0.07 A W^−1^ at 560 nm (Fig. S25). This non-monotonic behavior aligns precisely with the absorption characteristics predicted by our optical simulations, confirming the critical importance of layer thickness optimization for achieving photocurrent matching in tandem devices. In comparison to the single photodiode with the same 400 nm PbSe CQDs layer, the dark and light *J*-*V* curves in Figs. 3a and S26 show that the tandem photodiodes have a similar photo-response to 1550 nm light with the single photodiode. The tandem photodiodes exhibit an EQE of 25.06%, which is similar to 25.47% of the single photodiode under the same light power density (400 μW cm^−2^). This performance parity provides direct experimental evidence of effective photocurrent matching between the front and rear junctions at the target wavelength. The photo-voltage of the tandem device under the condition of *J* = 0 is about twice higher than the single photodiode, indicating the excellent transportation between the front and rear junctions without an interfacial barrier.

We further characterized the spectral response of the single photodiode and tandem photodiodes under modulated light from LEDs with different wavelengths (365–1650 nm) at a fixed power density of 400 μW cm^−2^. The current density-time (*J*-*T*) curves and *J*-*V* curves in Figs. 3b and S21 show that the tandem photodiodes have suppressed photo-response to visible light compared with the single photodiode. The EQE statistics of 6 single photodiodes and tandem photodiodes to different wavelengths are collected in Fig. 3c. Specifically, the tandem devices exhibit an average EQE of approximately 7% across the visible spectrum—representing a 13-fold reduction compared to the 90% EQE measured for single photodiodes. Crucially, this enhanced spectral selectivity is achieved without compromising performance at the target wavelength, as the tandem photodiodes maintain an EQE of 25.06% at 1550 nm, equivalent to that of single-junction devices. We also compared a single photodiode with a CQDs filter to the tandem photodiode (Fig. S27). Both configurations exhibit comparable spectral selectivity, significantly surpassing the unfiltered single junction. These experimental results quantitatively validate the FDTD simulations and confirm that the tandem architecture effectively minimizes interference from visible background radiation while preserving high sensitivity at the operational wavelength of 1550 nm.

The linear dynamic range (LDR) represents the detector’s ability to accurately convert variations in input signals into output signals without saturation or distortion^53,54^. The *J*-*V* curves of the tandem photodiodes are shown in Fig. 3d under 1550 nm illumination with different power densities from 2.40 × 10^−5^ to 8.04 mW cm^−2^. The LDR of the tandem photodiodes is over 99.6 dB at −0.4 V as illustrated in Fig. 3e attributed to efficient collection of photo-generated carriers, which originated from the high carrier mobility of PbSe CQDs (Fig. S6) and the good band alignment (Fig. S12) in the device. Figure 3f illustrates the noise current spectrum (100 Hz-51.3 kHz) of the tandem photodiodes. We picked the noise current of 3.63 × 10^−13^ A Hz^−1/2^ at 1 kHz for the specific detectivity (D^*^) calculation. The D^*^ of the tandem PbSe CQDs photodiodes is about 2.29 × 10^11^ Jones, which is comparable with the reported PbS CQDs photodiodes at 1550 nm^55–58^.

**Fig. 4:**
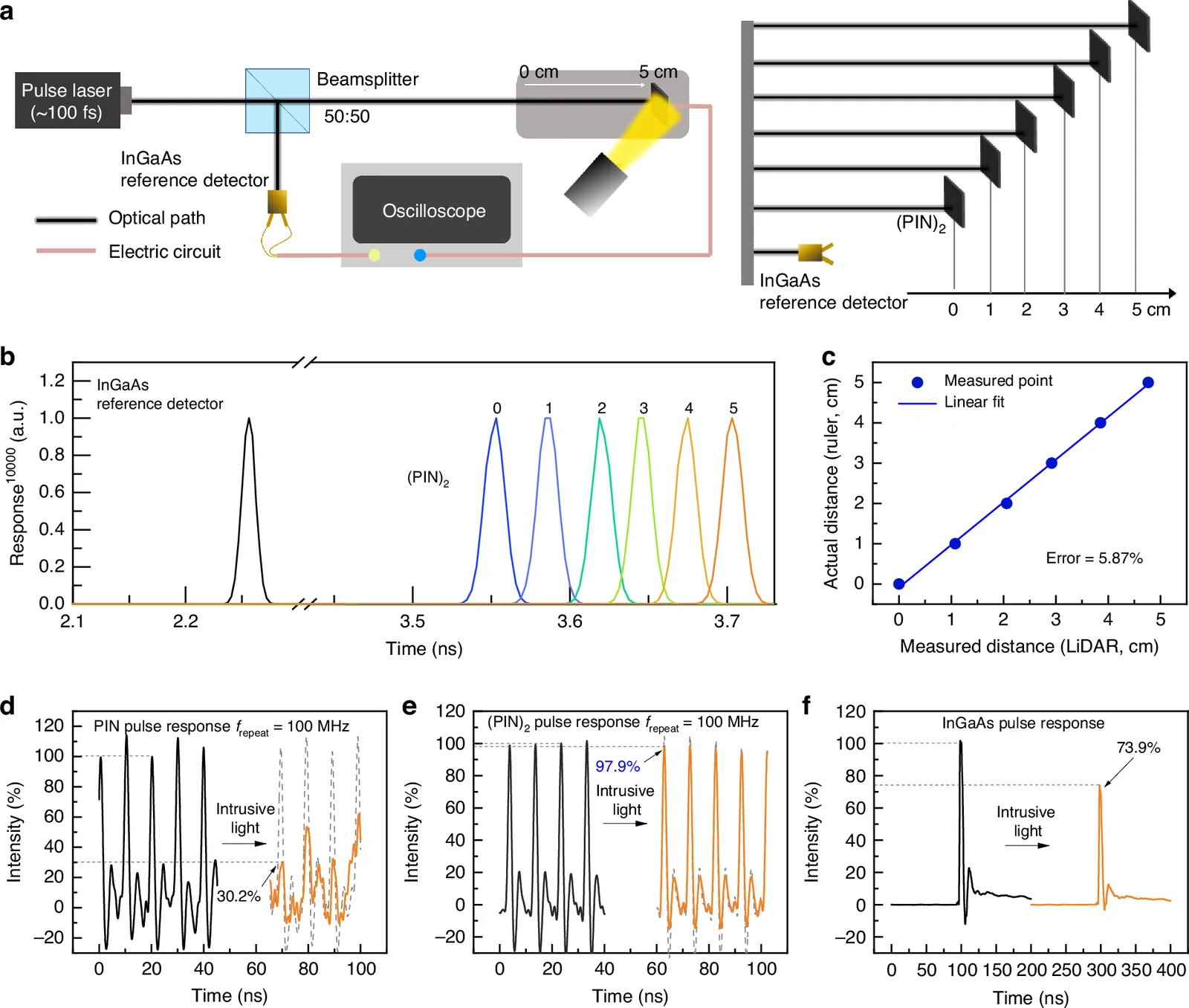
LiDAR measurements based on the tandem CQDs photodiodes. **a** Schematic diagram of the LiDAR measurement. **b** Pulse laser response spectra of the reference InGaAs detector and the tandem photodiodes at sites from 0 to 5 cm. **c** Ruler-measured distance versus LiDAR-measured distance. Pulse laser response of **d** the single photodiode, **e** the tandem photodiodes, and **f** the InGaAs photodiode with or without an intrusive tungsten halogen lamp

We implemented a LiDAR system to evaluate the depth-sensing capabilities of the tandem CQDs photodiodes, as schematically illustrated in Figs. 4a and S28. The pulse laser response spectra of the tandem photodiodes were recorded for every precise 1 cm displacement, and the overall response spectra with varying distances are illustrated in Figs. 4b and S29. We performed a first-order derivative on the response spectra, as shown in Fig. S30, to use the zero-crossing difference as the time difference. We employed time-correlated single-photon counting (TCSPC) method (1024 points at each distance) to mitigate testing system errors and acquire more accurate time differences at each light travel distance. This approach successfully achieved the ranging error of 5.87% at centimeter-level resolution ranging, as shown in Fig. 4c and Fig. S31. Additionally, we assembled a 1 × 6 mirror array (Fig. S32) to visualize the accuracy of the depth measurement using the tandem photodiodes. The 3D image in Fig. S33 shows that the tandem photodiodes provide a centimeter-level resolution for LiDAR.

To evaluate the spectral selectivity of the tandem photodiodes under realistic operating conditions, we compared their 1550 nm pulsed laser response against single-junction CQD devices and commercial InGaAs photodiodes when subjected to broadband white light interference. The experimental setup simulated practical LiDAR scenarios where filtered background illumination persists, as detailed in Note S4. Figure 4d–f present the measured scatter-back signal intensity with or without the optical filtered interference light. Under the interference of white light, the signal intensity of the single photodiode is degraded to 30.2% of the original intensity due to the saturation of photo-generated carriers as shown in Fig. 4d. At the same interference condition, the tandem photodiodes keep almost the same signal intensity (97.9% in Fig. 4e), which is even better than 73.9% of the InGaAs photodiode (Fig. 4f). These results quantitatively validate the exceptional interference rejection capability of our tandem architecture and highlight its potential as a competitive alternative for robust 1550 nm LiDAR systems operating under challenging ambient light conditions.

## Discussion

In conclusion, we have successfully demonstrated tandem PbSe CQDs photodiodes as a LiDAR detector. The optimized devices achieve a record-fast response time of 1.01 ns, an extensive LDR of 99.6 dB, a high detectivity of 2.29 × 10^11^ Jones at 1550 nm, and a 13-fold suppressed EQE in the interference visible range. In a functional LiDAR system, the tandem photodiodes enable centimeter-level spatial resolution while maintaining 97.9% of the signal-to-noise ratio under strong background illumination. This exceptional performance stems from the tandem architecture’s dual mechanism: the series connection inherently halves the junction capacitance to accelerate response speed, while the photogenerated carrier mismatch between front and rear junctions effectively suppresses visible-light interference through charge collection narrowing. Future development focusing on advanced surface passivation strategies for PbSe CQDs is expected to further reduce dark current and enhance the practical deployment of tandem photodiodes. Collectively, this work establishes a new architectural paradigm for cost-effective 1550 nm LiDAR technology, creating opportunities for widespread adoption in consumer electronics and autonomous driving systems.

## Materials and methods

### PbSe CQDs ligand exchange and film deposition

PbSe CQDs were synthesized using the reported cation exchange method^59^ and dispersed in octane with a concentration of 6 mg mL^−1^ as CQDs solution. 920 mg PbI_2_ and 580 mg PbBr_2_ were dissolved in 10 mL N, N-dimethylformamide (DMF) as ligand solution. 10 mL CQDs solution was mixed with 10 mL ligand solution (PbBr_2_ and PbI_2_ in DMF solution) and subsequently shaken for 40 s. CQDs were all transferred from octane to DMF and capped with halides, and the upper octane was removed. 10 mL octane was then added and shaken for 40 s to remove residual oleic acid. This purification procedure was repeated twice. The CQDs in DMF were centrifuged at 9000 rpm for 5 min and dried in vacuum for 40 min. The halide-capped CQDs powder was re-dispersed with a concentration of 100–600 mg mL^−1^ in DMF and butylamine mixture (1:1 volume ratio) as CQDs ink. The CQDs ink was spin-coated onto substrates at 2500 rpm for 40 s to form CQDs film. The CQDs film was annealed at 90 °C for 10 min to remove residual solvent. All experimental procedures were conducted in nitrogen.

### Device fabrication

The device was fabricated on a 2.5 cm × 2.5 cm glass coated with 180 nm indium tin oxide (ITO) film. A 200 nm ZnO film was deposited on the ITO substrate by magnetron sputtering at a RF mode with a power of 200 W. Halide-capped PbS CQDs ink was prepared with a concentration of 25–100 mg mL^−1^ according to the above ligand exchange procedures. After deposition of halide-capped PbSe CQDs film, the device was again annealed in vacuum at 70 °C for 30 mins. Then the halide-capped PbS CQDs ink was spin-coated at 2500 rpm for 40 s and annealed at 90 °C for 10 min to form a grading layer. The annealing has almost no effect on the device performance, as shown in Fig. S34. PbS-EDT layer was spin-coated layer by layer according to the reported method^52^. 1 nm Au film was thermally evaporated at the rate of 0.5 Å s^−1^ as an intermediate layer. 7 nm C_60_ was thermally evaporated to protect CQDs layer from the damage of subsequent RF sputtered ZnO. The upper CQDs junction was fabricated by the same procedures. 70 nm Au film was thermally evaporated and patterned by a shadow mask as top electrodes.

### Material characterizations

The absorption spectra were measured by a SolidSpec-3700 ultraviolet-visible-near infrared spectrophotometer. The cross-section SEM image was carried out by acquired using GeminiSEM 300 field emission. The film thickness was measured by the Bruker DektakXT stylus profilometer. The photoluminescence spectra were excited by a 980 nm laser and acquired by the QuantaMaster 8000 steady-state transient modular fluorescence spectrometer. The refractive index and extinction coefficient of the PbSe CQDs film were measured by the J. A. Woollam RC2 ellipsometer.

The transfer characteristic curves of the FET were acquired in nitrogen by an Agilent B1500A semiconductor characterization system. The mobility (*μ*) of the PbSe CQDs film is extracted from the transconductance (*g*_m_) as below

1 $$\mu =\frac{{g}_{{\rm{m}}}}{{C}_{{\rm{i}}}{V}_{{\rm{ds}}}}\times \frac{L}{W}$$

where the width-to-length ratio ($\frac{W}{L}$) of FET is 480, the source-drain voltage (*V*_ds_) is 0.5 V, and the capacitance per unit area of SiO_2_ insulating layer (*C*_i_) is 1.15 × 10^−8^ F cm^−2^. The conductivity (*δ*) of the PbSe CQDs film was acquired from the output characteristic curves

2 $$\delta =\frac{I\times l}{(V\times S)}$$

where *I* is the measured current, *l* is the length of the channel, *V* is the applied voltage, and *S* is the cross-sectional area of the channel. The carrier concentration (*n*) of the PbSe CQDs film was calculated as below

3 $$n=\frac{\delta }{q\mu }$$

where *q* is the electronic charge.

The THz transmission spectrum of the PbSe CQDs film was measured using QT-TS1000. The complex conductivity ${\tilde{\sigma }}_{0}$ was fitted using the Lorentz model as shown below^60^.

4 $${\tilde{\sigma }}_{0}=A\frac{i\omega }{{\omega }^{2}-{\omega }_{{\rm{L}}}^{2}+i\frac{\omega }{\tau }}$$

where *A* is the amplitude, *ω* is the THz frequency, *ω*_L_ is the resonance frequency, and *τ* is the electron scattering time. The mobility can be calculated using the following expression.

5 $$\mu =\frac{q\tau }{{m}^{\ast }}$$

where *q* is the elementary charge, m* is the electron effective mass of PbSe CQDs as 0.078 × *m*_0_, *m*_0_ is the electron mass^61^.

The *J*-*V* curves for the SCLC method were acquired by the Agilent B1500A semiconductor characterization system. Both linear and quadratic regions in the current−voltage curves are observed in Fig. S6c, which justifies the SCLC analysis. By analyzing the square-law region, the injected dark current density can be derived by the Mott−Gurney law.

6 $$J=\frac{9}{8}\frac{{\varepsilon }_{0}{\varepsilon }_{{\rm{r}}}\mu }{{L}^{3}}{V}^{2}$$

where *J* is the current density, *ε*_0_ is the vacuum permittivity, *ε*_r_ is the relative dielectric constant of PbSe CQDs film, *μ* is the mobility of PbSe CQDs film, *L* is the thickness of PbSe CQDs film, and *V* is the applied voltage.

### Photoelectric characterizations

The pulse laser response was performed by using the LH-CRPS probe station, the CARBIDE-CB3-40W 1030 nm fs pulse laser, and Keysight DSO-S 054 A oscilloscope. The probes were directly connected to the oscilloscope with 50 Ω input BNC cables. All the response time measurements were carried out without external bias across devices. The repetition rate of the pulse laser was set as 4 kHz.

The *C*-*f* spectra were measured by the Agilent 4294 A. The Agilent 4294 A was set at *C*_p_-*R*_p_ mode with carefully open-circuit and short-circuit calibration. The frequency range was set from 80 Hz to 1 MHz with the AC oscillator level of 30 mV.

The *J*-*V* curves were acquired by the Agilent B1500A semiconductor characterization system. The light sources were Thorlabs LEDs with central wavelengths of 365, 490, 590, 780, 970, 1200, 1550, and 1650 nm. The power density was calibrated by a power meter (PM101, Thorlabs). The responsivity (*R*) was calculated as below

7 $$R=\frac{{J}_{{\rm{p}}}-{J}_{{\rm{d}}}}{P}$$

where *J*_p_ is the photocurrent density, *J*_d_ is the dark current density, and *P* is the power density of light. The EQE was calculated as below

8 $${\rm{EQE}}=\frac{1.24\times R}{\lambda }$$

where λ is the wavelength of the light source.

The *I*-*T* curves were recorded by the Agilent B1500A semiconductor characterization system. The LEDs were modulated at 0.1 Hz by a waveform generator (Agilent 33611 A), and the power density was calibrated with a Thorlabs optical power meter.

The LDR was acquired from *J*-*V* curves under illumination with different power densities. The LED power density was calibrated from 24 nW cm^−2^ to 8.04 mW cm^−2^ by a power meter (PM101, Thorlabs). The LDR was calculated by the formula as below

9 $${\mathrm{LDR}}=20\log (\frac{{J}_{\max }}{{J}_{\min }})$$

where *J*_max_ and *J*_min_ were the maximum and minimum photocurrent density, which is linear to power density.

The noise current spectra were obtained by using a preamplifier (Stanford Research Systems, SR570) and a lock-in amplifier (Stanford Research Systems, SR850). Devices were connected to the preamplifier with the BNC cable and placed in a dark box to prevent environmental interference. The specific detectivity (D^*^) is calculated as below

10 $${D}^{\ast }=\frac{R\sqrt{A\Delta f}}{{i}_{{\rm{n}}}}$$

where *A* is the device electrode area, Δ*f* is the electrical bandwidth (1 Hz), *i*_n_ is the measured noise current in Fig. 3f.

### LiDAR measurements

The LiDAR measurements were completed by using a 1550 nm fs pulse laser, an InGaAs photodiode (LightSensing LSIPD-A75), a QVF135 interference light source, and the Keysight DSO-S 054 A oscilloscope. The pulse laser was split by a 50:50 beam splitter to the reference InGaAs photodiode and CQDs photodiodes. The CQDs device was placed on the displacement platform for its spatial movement. The oscilloscope was used for reading signals from the reference detector and CQDs device. These detectors were connected to the oscilloscope using gold-plated BNC cables and gold wires to ensure optimal transmission. The LiDAR demo was performed by recording the pulse laser response of the reference detector and CQDs device while moving the CQDs device finely from 0 to 5 cm. The distance *L* is calculated as below

11 $$L=\frac{c\times \Delta t}{2}$$

where c is the light speed, Δ*t* is the time difference.

### FDTD simulations

The FDTD simulations (in 2D mode) were conducted on stacked layers of the CQDs photodiodes in X and Y directions. The plane-wave source with a wavelength ranging from 0.3 to 2 μm was set to incident from the ITO side. The measured refractive index and extinction coefficient were used in the simulation. The transmission monitor was employed at the interface between the PbSe CQDs layer and other functional layers.

## Supplementary information

Supporting Information

Dataset

## Data Availability

The data that support the findings of this study are available from the figures and from the corresponding authors on reasonable request.
