# Supplementary material for: Spectral-selective light detection and ranging at 1550 nm using tandem colloidal quantum dot photodiodes
Source: Light Sci Appl. 2026 Aug 20;15:355. doi: 10.1038/s41377-026-02244-2 (PMC13493946; doi:10.1038/s41377-026-02244-2)
Supplement: Supplementary file 1 [file 41377_2026_2244_MOESM1_ESM.docx]

**Supplementary Information** **for**

**Spectral-selective Light Detection and Ranging at 1550 nm using Tandem Colloidal Quantum Dot Photodiodes**

Junrui Yang^1,2,3,4,5^, Jing Liu^1,2,3,4,5*^, Chengjie Deng^1,6^, Shuaicheng Lu^1,2^, Hao Ding^1^, Xing Zhou^7^, Long Hu^8^, Xinzheng Lan^1,3^, Jianbing Zhang^1,4,6^, Xing Yang^2^, Yihua Hu^2*^, Jiang Tang^1,3^ and Liang Gao^1,3,4,6*^

^1^ Wuhan National Laboratory for Optoelectronics (WNLO) and School of Optical and Electronic Information, Huazhong University of Science and Technology, 1037 Luoyu Road, 430074 Wuhan, China.

^2^ State Key Laboratory of Pulsed Power Laser Technology, 460 Huangshan Road, 230037 Hefei, China.

^3^ Optics Valley Laboratory, 1037 Luoyu Road, 430074 Wuhan, China.

^4^ Wenzhou Advanced Manufacturing Technology Research Institute of Huazhong University of Science and Technology, 225 Chaoyang New Street, 325105 Wenzhou, China.

^5^ JFS Laborotory, 9 Jiulonghu Street, 430078 Wuhan, China.

^6^ Shenzhen Huazhong University of Science and Technology Research Institute, 9 Yuexingsan Road, 518057 Shenzhen, China.

^7^ School of Materials Science and Engineering, Huazhong University of Science and Technology, 1037 Luoyu Road, 430074 Wuhan, China.

^8^ School of Materials Science and Engineering, The University of New South Wales, Sydney, NSW, 2052, Australia.

* Corresponding Author: Jing Liu (jingj@hust.edu.cn), Yihua Hu (yh_hu@263.com), Liang Gao (highlight@hust.edu.cn)

**Note S1|** Calculation of the intensities of interference sunlight and scatter-back signal light.

To calculate the intensity of the scatter-back signal light, we used the commonly used equation in LiDAR system. This equation describes the relation of scatter-back signal intensity versus distance^1^. It can be expressed as below at the situation that the size of the target is larger than the laser spot.

$P_{\text{R}}=\frac{\text{π}P_{\text{T}}\rho_{\text{Ext}}D^{2}}{(4R)^{2}}\eta_{\text{Atm}}\eta_{\text{Sys}}$ (S1)

where *P*_R_ is the power of reflective signal, *P*_T_ is the power of emitted signal, *ρ*_Ext_ is the reflectivity of target, *D* is the aperture diameter of detector, *R* is the distance between target and LiDAR, *η*_Atm_ is the atmospheric transmission coefficient, and *η*_Sys_ is the transmission coefficient of optical system.

Assuming the scenario where the LiDAR needs to detect an object at 250 m ahead under favorable weather conditions (relative humidity of 50% and visibility of 5 km), the *η*_Atm_ is about 0.89 using the formula *η*_Atm_ = exp(−2*γR*), where *γ* is atmospheric extinction coefficient equaling 0.227 km^−1^ as reported in the reference^4^. Commonly reported parameters are used for this calculation: the *P*_T_ is 10 kW;^2^ the *ρ*_Ext_ is assumed to be 10%;^4^ the *D* is 100 μm, matching the electrode size of our best tandem photodiode; the *R* is 250 m; the *η*_Sys_ is 1 under ideal condition. Based on these parameters, the calculated power of scatter-back signal light is 27.9 pW.

For the optical filter FBH1550-30 (OD>5), the leaked power density of AM1.5G sunlight is 8.72×10^−8^ W cm^−2^ ranging from 400 to 1400 nm. With an active electrode area of 7.85×10^−3^ cm^2^ (the *D* is 100 μm), the corresponding leaked sunlight power is about 684.5 pW on the detector. The intensity of leaked interference sunlight is about 24.5 times greater than the scatter-back signal light under AM1.5G.

Furthermore, we calculated the sunlight intensity under varying weather conditions, including cloudy skies (cloud cover ranging from 40% to 90% of the sky dome) and overcast skies (cloud cover exceeding 90%) from the solar spectra under different weathers as shown in Fig. S2^3^. Under overcast condition, the leaked sunlight power on the active device area is 111.35 pW, while for cloudy conditions, the leaked sunlight power is 283.39 pW. Under the 25 mm h^−1^ rainfall combined with overcast sky conditions, the atmospheric extinction coefficient significantly increases to 15.89 km, resulting in a calculated *η*_Atm_ of 0.0188 and a reflective signal intensity (*P*_R_) of 0.59 pW. In this case, the leaked sunlight intensity is approximately 188.73 times higher than the reflective signal.

In the case of extreme foggy weather with a visibility of 0.5 km, the corresponding atmospheric extinction coefficient is 45.4 km^−1^. The calculated *η*_Atm_ is 1.18×10^−5^ and calculated *P*_R_ is 0.37 fW. The leaked sunlight is approximately 3×10^5^ times greater than *P*_R_.

**Note S2|** Response time modeling of single-junction photodiode (called as PIN).

The response time (*τ*) of single photodiode is composed of the transit time (*τ*_transit_) and the RC time (*τ*_RC_), and can be expressed as below

$\tau=\sqrt{\left(\tau_{\text{transit}}\right)^{2}+\left(2.2\times\tau_{\text{RC}}\right)^{2}}$ (S2)

The transit time describes the time of photo-carriers transiting to electrodes, and can be expressed as below

$\tau_{\text{transit}}=\frac{d^{2}}{\mu V}$ (S3)

where *d* is the depletion region thickness, *μ* is the CQDs film mobility, *V* is the built-in voltage. The RC time can be expressed as

$\tau_{\text{RC}}=RC=\frac{R\varepsilon_{0}\varepsilon_{\text{r}}A}{d}$ (S4)

where *R* is the resistance primarily determined by the impedance of the external testing circuit (50 Ω), *C* is the capacitance in the measurement circuit (mainly junction capacitance), *ε*_0_ the vacuum permittivity, *ε*_r_ is the relative permittivity, and *A* is the active area.

**Note S3|** Deduction of tandem photodiodes (called as (PIN)_2_) model.

To attain a comprehensive understanding of the tandem photodiodes, we conducted an extensive analysis of the equivalent circuit model depicted in Fig. S5. Here, we defined a new parameter *γ* meaning mismatch of capacitance between two junctions as follows

$\gamma=\frac{C_{\text{j2}}}{C_{\text{j1}}}-1=\frac{d_{1}}{d_{2}}-1$ (S5)

where *C*_j1_ and *d*_1_ are the capacitance and depletion region width of the front junction respectively, *C*_j2_ and *d*_2_ are the capacitance and depletion region width of the rear junction.

Firstly, we calculated the admittance (*Y*) of equivalent circuit of tandem model as illustrated in Fig. S5

$Y=\frac{Y_{1}\times Y_{2}}{Y_{1}+Y_{2}}$ (S6)

where *Y*_1_ and *Y*_2_ are the admittance of the front and rear junctions respectively. The admittance can be expressed as

$Y=\frac{\left(\frac{1}{R_{\text{sh1}}}+j\omega C_{\text{j1}}\right)\left(\frac{1}{R_{\text{sh2}}}+j\omega C_{\text{j2}}\right)}{\left(\frac{1}{R_{\text{sh1}}}+\frac{1}{R_{\text{sh2}}}+j\omega(C_{\text{j1}}+C_{\text{j2}})\right)}$ (S7)

where *j* is the imaginary unit, *ω* is the angular frequency of input signal, *R*_sh1_ is the shunt resistance of the front junction, *R*_sh2_ is the shunt resistance of the rear junction. We multiplied $\left(\frac{1}{R_{\text{sh1}}}+\frac{1}{R_{\text{sh2}}}-j\omega(C_{\text{j1}}+C_{\text{j2}})\right)$ both on numerator and denominator to eliminate the disturbance of *j*. The denominator can be expressed as:

$(\frac{1}{R_{\text{sh1}}}+\frac{1}{R_{\text{sh2}}})^{2}+\omega^{2}(C_{\text{j1}}+C_{\text{j2}})^{2}$ (S8)

The numerator can be expressed as:

$\left(\frac{1}{R_{\text{sh1}}}+j\omega C_{\text{j1}}\right)\left(\frac{1}{R_{\text{sh2}}}+j\omega C_{\text{j2}}\right)\left(\frac{1}{R_{\text{sh1}}}+\frac{1}{R_{\text{sh2}}}-j\omega(C_{\text{j1}}+C_{\text{j2}})\right)$ (S9)

Then the numerator can be simplified as:

$\left((\frac{1}{R_{\text{sh1}}R_{\text{sh2}}}-\omega^{2}C_{\text{j1}}C_{\text{j2}})+j\omega(\frac{C_{\text{j1}}}{R_{\text{sh2}}}+\frac{C_{\text{j2}}}{R_{\text{sh1}}})\right)\left(\frac{1}{R_{\text{sh1}}}+\frac{1}{R_{\text{sh2}}}-j\omega(C_{\text{j1}}+C_{\text{j2}})\right)$ (S10)

The imaginary part is related to equivalent capacitance and shown as below

$j\omega\left((\frac{C_{\text{j1}}}{R_{\text{sh2}}}+\frac{C_{\text{j2}}}{R_{\text{sh1}}})(\frac{1}{R_{\text{sh1}}}+\frac{1}{R_{\text{sh2}}})-(\frac{1}{R_{\text{sh1}}R_{\text{sh2}}}-\omega^{2}C_{\text{j1}}C_{\text{j2}})(C_{\text{j1}}+C_{\text{j2}})\right)$ (S11)

*C*_j1_ and *C*_j2_ can be expressed as below

$\left\{\begin{matrix} C_{\text{j}1}=\frac{\varepsilon_{0}\varepsilon_{\text{r}}A}{d_{1}} \\ C_{\text{j2}}=\frac{\varepsilon_{0}\varepsilon_{\text{r}}A}{d_{2}} \end{matrix}\right.$ (S12)

where *ε*_0_ is the vacuum permittivity, *ε*_r_is the relative dielectric constant of PbSe CQDs film, *A* is the electrode area, *d*_1_ is the front depletion region width, *d*_2_ is the rear depletion region width. Under full depletion condition, *d*_1_ is the front PbSe CQDs film thickness and *d*_2_ is the rear PbSe CQDs film thickness. The corresponding front shunt resistance *R*_sh1_ and rear shunt resistance *R*_sh2_ can be expressed as below.

$\left\{\begin{matrix} R_{\text{sh1}}=\frac{\rho d_{1}}{A} \\ R_{\text{sh2}}=\frac{\rho d_{2}}{A} \end{matrix}\right.$ (S13)

where *ρ* is the resistivity. We take the expression of *C*_j1_, *C*_j2_, *R*_sh1_, *R*_sh2_ into equation S10 and the simplified equation is shown as below

$\left\{\begin{matrix} (\frac{C_{\text{j1}}}{R_{\text{sh2}}}+\frac{C_{\text{j2}}}{R_{\text{sh1}}})(\frac{1}{R_{\text{sh1}}}+\frac{1}{R_{\text{sh2}}})=\frac{2\varepsilon_{0}\varepsilon_{\text{r}}A^{3}}{\rho^{2}}\times\frac{d_{1}+d_{2}}{d_{1}^{2}d_{2}^{2}} \\ (\frac{1}{R_{\text{sh1}}R_{\text{sh2}}}-\omega^{2}C_{\text{j1}}C_{\text{j2}})(C_{\text{j1}}+C_{\text{j2}})=(\frac{\varepsilon_{0}\varepsilon_{\text{r}}A^{3}}{\rho^{2}}-\omega^{2}(\varepsilon_{0}\varepsilon_{\text{r}})^{3}A^{3})\times\frac{d_{1}+d_{2}}{d_{1}^{2}d_{2}^{2}} \end{matrix}\right.$ (S14)

The simplified imaginary part of numerator is shown below.

$(\frac{\varepsilon_{0}\varepsilon_{\text{r}}A^{3}}{\rho^{2}}+\omega^{2}(\varepsilon_{0}\varepsilon_{\text{r}})^{3}A^{3})\times\frac{d_{1}+d_{2}}{d_{1}^{2}d_{2}^{2}}$ (S15)

The equivalent capacitance (*C*_eq_) can be expressed as:

$C_{\text{eq}}=\frac{(\frac{\varepsilon_{0}\varepsilon_{r}A^{3}}{\rho^{2}}+\omega^{2}(\varepsilon_{0}\varepsilon_{r})^{3}A^{3})\times\frac{d_{1}+d_{2}}{d_{1}^{2}d_{2}^{2}}}{(\frac{1}{R_{\text{sh1}}}+\frac{1}{R_{\text{sh2}}})^{2}+\omega^{2}(C_{\text{j1}}+C_{\text{j2}})^{2}}$ (S16)

Taking the expression of *C*_j1_, *C*_j2_, *R*_sh1_, *R*_sh2_ to simplify.

$C_{\text{eq}}=\frac{(\frac{\varepsilon_{0}\varepsilon_{\text{r}}A^{3}}{\rho^{2}}+\omega^{2}(\varepsilon_{0}\varepsilon_{\text{r}})^{3}A^{3})\times\frac{d_{1}+d_{2}}{d_{1}^{2}d_{2}^{2}}}{(\frac{A^{2}}{\rho^{2}}+\omega^{2}(\varepsilon_{0}\varepsilon_{\text{r}})^{2}A^{2})\times(\frac{1}{d_{1}}+\frac{1}{d_{2}})^{2}}=\frac{\varepsilon_{0}\varepsilon_{\text{r}}A}{(d_{1}+d_{2})}=\frac{\varepsilon_{0}\varepsilon_{\text{r}}A}{(\gamma+2)d_{2}}$ (S17)

Considering the condition of *d*_1_ ≥ *d*_2_ (*γ* ≥ 0), we bring *C*_eq_ into equation S1. The transit time is determined by the wider *d*_1_ junction due to the synchronous in *d*_1_ and *d*_2_ junction as shown in Fig. 1e

$\tau=\sqrt{\left[\frac{\varepsilon_{0}\varepsilon_{\text{r}}A}{\left(\gamma+2\right)d_{2}}\times R\right]^{2}+\left[\frac{\left(\gamma+1\right)^{2}d_{2}^{2}}{\mu V}\right]^{2}}\left(\gamma\geq0\right)$ (S18)

We conduct the equation S18 as simplified equation S17 to solve the response time at the optimized thickness *d*_2_.

$f\left(d_{2}\right)=\tau^{2}=\frac{m}{d_{2}^{2}}+n\times d_{2}^{4}$ (S19)

where the *m* is a parameter proportional to (*γ*+2)^−2^, *n* is a parameter proportional to (*γ*+1)^4^, and neither of them depends on thickness *d*_2_. We solve equation S18 and get that *f*(*d*_2_) is minimized when $d_{2}=\left(\frac{m}{2n}\right)^{1/6}$. The corresponding minimum *f*(*d*_2_) is as below

$f\left(\left(\frac{m}{2n}\right)^{1/6}\right)=\left(2^{-2/3}+2^{1/3}\right)m^{2/3}n^{1/3}$ (S20)

We substituted *m* and *n* into the equation S19 and found the $f\left(\left(\frac{m}{2n}\right)^{1/6}\right)$ is proportional to (*γ*+1)^4/3^/(*γ*+2)^4/3^*.*

$f\left(\left(\frac{m}{2n}\right)^{1/6}\right)\propto\left(\frac{\gamma+1}{\gamma+2}\right)^{4/3}=[1-\frac{1}{\gamma+2}]^{4/3}$ (S21)

Here, $f\left(\left(\frac{m}{2n}\right)^{1/6}\right)$ attains its minimum value at *γ* = 0 (*γ* ≥ 0), indicating the shortest response time.

Considering the other scenario where *d*_1_ ≤ *d*_2_(−1 ≤ *γ* ≤ 0), the $f\left(\left(\frac{m}{2n}\right)^{1/6}\right)$ is expressed as below

$f\left(\left(\frac{m}{2n}\right)^{1/6}\right)\propto\frac{1}{(\gamma+2)^{4/3}}$ (S22)

$f\left(\left(\frac{m}{2n}\right)^{1/6}\right)$ also attains its minimum value at *γ* = 0. The case of *γ* = 0 corresponds to the situation where the depletion region thickness of front and rear PIN junction is the same. Therefore, the response time of (PIN)_2_ structure at the optimized thickness *d*_2_ is minimized only when the capacitance of the front PIN junction equals that of the rear PIN junction.

**Note S4|** Spectral-selective ability measurements of PIN, (PIN)_2_ and InGaAs detectors.

The spectral-selective measurements were conducted by reproducing the signal light and filtered interference light results calculated in **Note S1**. The signal light and filtered interference light were optically amplified proportionally to enable the observation on oscilloscope. The final incident signal light intensity was amplified to 3 kW cm^−2^ (10 ps pulse width, 30 mW peak power, 100 MHz repetition rate), with the interference light amplified to 158 mW cm^−2^. Considering energy conservation between the light pulse (10 ps) and pulse response (1.01 ns), this results in a peak attenuation factor of 1.01 ns/10 ps = 101 for tandem photodiodes and 1.69 ns/10 ps = 169 for single photodiodes. The equivalent signal intensity is thus 181.8-fold of interference light intensity during the measurements, corresponding to the actual scenario of 27.9 pW signal light and 176.1 fW filtered interference light. The laser pulse response of PIN, (PIN)_2_, and InGaAs detectors are shown in Fig. 4d-f.

**Fig. S1|** Absorption coefficient of PbSe CQDs film with an exciton absorption peak around 1550 nm.

**Fig. S2|** Solar spectra under different weathers.^3^

**Fig. S3|** LiDAR system using single photodiode (PIN device).

**Fig. S4|** Evolution of detectors from a component to an integrated system. Schematic diagram of **(a)** a standard single-junction detector, **(b)** a filtered detector as an assembly of discrete parts, and **(c)** our tandem architecture that reconfigures the entire photonic-electronic link.

**Fig. S5|** Equivalent circuit models of single photodiode and tandem photodiodes.

**Fig. S6| Measured mobility of PbSe CQDs film. a,** Transfer characteristic curve of PbSe CQDs field-effect transistor (FET). **b,** Complex conductivity and fitted curves of PbSe CQDs film acquired from THz spectrum. **c,** *J*-*V* curves measured in space-charge limited current (SCLC) method. The measured mobility is 0.2121 cm^2^ V^−1^ s^−1^ for FET method, 3.65 cm^2^ V^−1^ s^−1^ for THz spectrum method and 1.59 cm^2^ V^−1^ s^−1^ for SCLC method.

**Fig. S7|** Carrier concentrations of halide-capped PbSe CQDs films stored in nitrogen and in air.

**Fig. S8|** Mobility of halide-capped PbSe CQDs films stored in nitrogen and in air.

**Fig. S9|** Dark current density-voltage (*J*-*V*) curves of PbSe CQDs junctions fabricated on **(a)** ethanedithiol (EDT) treated PbS CQDs, **(b)** SnO_2_, and **(c)** ZnO layers.

**Fig. S10|** Photoluminescence (PL) spectra of PbSe CQDs films fabricated on **(a)** EDT treated PbS CQDs, **(b)** SnO_2_ and ZnO layers.

**Fig. S11|** **Effect of PbS CQDs grading layer on single-junction PbSe CQDs photodiode. a,** Dark and light (370 μW cm^−2^ at 1550 nm) *J*-*V* curves of PbSe CQDs single photodiodes without PbS CQDs grading layers (a) and with varied PbS CQDs grading layers thicknesses of **(b)** 20 nm, **(c)** 40 nm, **(d)** 80 nm.

**Fig. S12|** Measured UPS results of **(a-b)** EDT-PbS CQDs layer, **(c-d)** halide-PbSe CQDs layer, **(e-f)** PbS CQDs grading layer and **(g-h)** ZnO layer. **i,** Energy band structure of PbSe CQDs single photodiode.

**Fig. S13|** Dark and light (370 μW cm^−2^ at 1550 nm) *J*-*V* curves of optimal single-junction PbSe CQDs photodiode.

**Fig. S14|** Designed shadow mask for top electrode deposition.

**Table S1| Thickness of PbSe CQDs films measured by step profiler.**

| Ink concentration (mg mL^−1^) | 100 | 200 | 500 | 650 |
| --- | --- | --- | --- | --- |
| Thickness (nm) | 74.50 | 208.10 | 398.11 | 562.23 |
|  | 75.47 | 217.14 | 401.21 | 554.31 |
|  | 76.73 | 209.31 | 410.68 | 557.02 |
|  | 72.68 | 212.36 | 405.68 | 565.88 |
| Average (nm) | 74.85 | 211.72 | 403.92 | 559.86 |

**Fig. S15|** Calculated response time (lines) and measured response time (dots) of (PIN)_2_ devices with varied electrode areas (3.14 mm^2^, 2.01 mm^2^, 0.50mm^2^, 0.13mm^2^, 0.03mm^2^) and varied thicknesses of CQDs film (560 nm, 400 nm, 200 nm, 75 nm) using the mobility measured by the SCLC method.

**Fig. S16|** Measured pulse laser responses of single-junction PbSe CQDs photodiodes with varied electrode areas (3.14 mm^2^, 2.01 mm^2^, 0.50mm^2^, 0.13mm^2^ and 0.03mm^2^).

**Fig. S17|** Measured capacitance-frequency (*C*-*f*) spectra of single-junction photodiode and tandem photodiodes with varied electrode areas of **(a)** 3.14 mm^2^, **(b)** 2.01 mm^2^, **(c)** 0.50 mm^2^, **(d)** 0.13 mm^2^, and **(e)** 0.03 mm^2^.

**Fig. S18|** Measured pulse laser responses of single-junction and tandem devices with an electrode area of 0.00785 mm^2^.

**Table S2| Comparison of device performance with previously reported studies.**

| Materials | Wavelength range (nm) | Response time (ns) | EQE | Detectivity (Jones) | Active area  (μm^2^) | References |
| --- | --- | --- | --- | --- | --- | --- |
| PbS CQDs | 350-1550 | 10 | 80% | 8×10^11^ | 7850 | [5] |
| PbS CQDs | 500-1370 | 200 | 65% | 2×10^11^ | 31416 | [6] |
| PbS CQDs | 400-1550 | 7 | 70% | 10^12^ | 10000 | [7] |
| PbS CQDs | <800-1330 | 4 | 42% | 3.9×10^11^ | 1962.5 | [8] |
| PbSe CQDs | 970-1550 | 1.01 | 25% | 2.29×10^11^ | 7850 | This work |
| PbSe CQDs | 970-1550 | 7.38 | 25% | 2.29×10^11^ | 31416 | This work |

**Fig. S19|** **Record performance unlocked by the tandem architecture.** Comparison of response time against active area highlights our tandem device as the fastest CQD photodiode at the same area (data are from Table S2).

**Fig. S20|** **a**, Pulse laser responses and **b**, Response time of optimal tandem photodiodes under varied laser power densities from 4.6 mW cm^−2^ to 0.3 mW cm^−2^.

**Fig. S21|** *J*-*V* curves of **(a)** single-junction CQDs photodiode and **(b)** tandem CQDs photodiodes under illumination with different wavelengths (365-1650 nm) and the same power density of 400 μW cm^−2^.

**Fig. S22| Optical parameters of halide-capped PbSe CQDs film. a**, Relative dielectric constant. **b,** Measured refractive index and **c**, Measured extinction coefficient by the ellipsometer method. The real part of the permittivity can be expressed as below

$$\varepsilon'=n^{2}-k^{2}$$

where *ε*’ is the relative dielectric constant (the real part of the permittivity), *n* is the refractive index, and *k* is the extinction coefficient.

**Fig. S23| FDTD simulations. a**, FDTD model of tandem photodiodes. **b**, Simulated absorption at 1550 nm of single photodiode, front junction and rear junction of tandem photodiodes with varied thicknesses of PbSe CQDs layer. **c**, Simulated absorption spectra of single photodiode, front junction and rear junction of tandem photodiodes with 412 nm PbSe CQDs layer. **d**, Simulated absorption at 1550 nm of front junction and rear junction of tandem photodiodes with varied angle of incident light. **e**, Simulated absorption at 400 nm of front junction and rear junction of tandem photodiodes with varied angle of incident light.

**Fig. S24|** Dark and light (370 μW cm^−2^ at 1550 nm) *J*-*V* curves of tandem photodiodes with varied PbSe CQDs layers thicknesses of **(a)** 75 nm, **(b)** 200 nm, **(c)** 400 nm, and **(d)** 560 nm.

**Fig. S25|** Responsivity of tandem photodiodes at 1550 nm with varied PbSe CQDs layers thicknesses.

**Fig. S26|** Dark and light (400 μW cm^−2^ at 1550 nm) *J*-*V* curves of single-junction photodiode and tandem photodiodes.

**Fig. S27|** Broadband EQE spectra of single-junction CQDs photodiode (with or without a CQDs filter) and tandem CQDs photodiodes at −0.4 V. The CQDs filter comprises of the same CQDs used as the active layer.

**Fig. S28| a**, Photo of built LiDAR system. **b**, Photo of measured signals on oscilloscope.

**Fig. S29|** Pulse laser responses of the reference detector and tandem photodiodes with varied distances (0, 1, 2, 3, 4, and 5 cm).

**Fig. S30|** First-order derivative of pulse laser responses of the reference detector and tandem photodiodes.

**Fig. S31|** Time difference distribution of light travel distance from 0 to 5 cm based on time-correlated single-photon counting (TCSPC) method.

**Fig. S32| a**, Top view and **b**, Oblique view of mirror array for depth measurements.

**Fig. S33|** A 3D visualization of the estimated depth using the peak position of the tandem photodiodes response.

**Fig. S34|** EQE variation of tandem photodiode annealing at 90 °C for 60 min.

**References**

1. Kashani, A.G., Olsen, M.J., Parrish, C.E., Wilson, N. A Review of LIDAR Radiometric Processing: From Ad Hoc Intensity Correction to Rigorous Radiometric Calibration. Sensors **15**, 28099-28128 (2015).
2. Zhang, Z., Li, Z., Yu, H., & Xuan, H. 10 kW peak power, single-frequency 1553 nm nanosecond pulsed fiber laser for time-of-flight LIDAR. Appl. Phys. Express **16**, 022002 (2023).
3. Del Pero, C., Aste, N., & Leonforte, F. The effect of rain on photovoltaic systems. Renew. Energy **179**, 1803-1814 (2021).
4. Wojtanowski, J., Zygmunt, M., Kaszczuk, M., Mierczyk, Z., & Muzal, M. Comparison of 905 nm and 1550 nm semiconductor laser rangefinders’ performance deterioration due to adverse environmental conditions. Opto-electron. Rev. **22**, 183-190 (2014).
5. Vafaie, M. et al. Colloidal quantum dot photodetectors with 10-ns response time and 80% quantum efficiency at 1,550 nm. Matter **4**, 1042–1053 (2021).
6. Atan, O. et al. Control over charge carrier mobility in the hole transport layer enables fast colloidal quantum dot infrared photodetectors. Nano Lett. **23**, 4298–4303 (2023).
7. Biondi, M. et al. Facet‐oriented coupling enables fast and sensitive colloidal quantum dot photodetectors. Adv. Mater. **33**, 2101056 (2021).
8. Deng, Y. et al. Short-wave infrared colloidal QDs photodetector with nanosecond response times enabled by ultrathin absorber layers. Adv. Mater. 2402002 (2024).
